# Accuracy of AI-assisted diagnostic tools for *Schistosoma haematobium*: A systematic review and meta-analysis

**DOI:** 10.1371/journal.pntd.0013703

**Published:** 2026-05-05

**Authors:** Sisay Desale, Getaneh Alemu, Tadesse Hailu

**Affiliations:** 1 Department of Medical Laboratory Science, Wollo University, Dessie, Ethiopia; 2 Department of Medical Laboratory Science, Bahir Dar University, Bahir Dar, Ethiopia; University of Buea, CAMEROON

## Abstract

**Background:**

Urogenital schistosomiasis caused by *Schistosoma haematobium* remains endemic in sub-Saharan Africa. Diagnosis traditionally relies on urine microscopy to detect parasite eggs; however, its sensitivity declines in low-intensity infections. Artificial intelligence (AI)-assisted image analysis offers a promising approach to automate egg detection and enhance diagnostic accuracy, but its performance compared with standard microscopy is not well established.

**Methods:**

We conducted a systematic review following the PRISMA guidelines and checklist. Studies evaluating AI-assisted detection of *S. haematobium* compared with microscopy and/or molecular reference standards, published up to August 2025, were identified through searches in PubMed/MEDLINE, HINARI, Epistemonikos, Science Direct, Google Scholar and grey literature sources. Eligible studies were selected based on pre-defined inclusion and exclusion criteria. The quality of included studies was assessed using the QUADAS-2 tool. Heterogeneity among studies was evaluated using the Cochrane Q test and I² statistic. Data was analyzed using STATA version 14.1 and Review Manager version 5.4.1.

**Results:**

Ten studies (15 datasets, 5,564 urine samples) conducted in sub-Saharan Africa met the inclusion criteria. AI-assisted tools demonstrated high diagnostic accuracy. The pooled sensitivity was 88% (95%CI 83%-91%) and pooled specificity was 89% (95% CI 83%-93%). The pooled diagnostic odds ratio was 54.00 (95% CI 30.41-95.88), indicating strong discrimination between infected and uninfected cases. The SROC curve yielded an AUC of 0.94 (95% CI 0.92-0.96), reflecting excellent overall accuracy. Heterogeneity across studies was high (I² = 100%), suggesting results varied by the specific AI platform and study context.

**Conclusion:**

AI-assisted microscopic diagnosis of *S. haematobium* achieved very good in this meta-analysis. These automated tools, whether smartphone-based or bench-top systems, showed promise for detecting infections and could help screen populations in endemic areas. With further validation in field settings and comparison to highly sensitive reference tests, AI diagnostic technology may become a valuable tool to improve case detection and support schistosomiasis control and elimination efforts.

## Author summary

Schistosomiasis (often called bilharzia) is a disease caused by a type of blood fluke worm that lives in freshwater snails. It affects millions of people, especially in poor tropical regions. *Schistosoma haematobium* is the species that causes urinary schistosomiasis. Infected people often suffer from blood in the urine, bladder problems, and kidney damage. Controlling schistosomiasis means finding and treating infected people. However, the usual way to diagnose it is by looking for parasite eggs under a microscope that can miss many infections, particularly when the number of eggs is low or lab facilities are limited. This makes it hard to reach goals for elimination of the disease. Artificial intelligence (AI) offers a new solution. Scientists have developed devices like smartphone microscopes or automated scanners that use AI programs to recognize parasite eggs in urine pictures. We collected and analyzed all published studies on these AI tools for detecting *S. haematobium*. We found that AI-based diagnostics are very accurate: on average they correctly identified 88% of infected cases and correctly ruled out about 89% of uninfected cases. In simpler terms, AI tools got nearly 9 out of 10 results right. Importantly, these tools work quickly and do not require highly trained experts. Our findings suggest that AI-assisted diagnosis could be a game-changer in low-resource settings. By making it easier and faster to screen for schistosomiasis, these technologies could help health workers find more infections that would otherwise be missed. Better detection means more people get treated, which lowers the spread of disease. In the long run, using AI tools for schistosomiasis could support global efforts to control and even eliminate this neglected tropical disease.

## Introduction

Schistosomiasis is a parasitic disease caused by trematodes of the genus *Schistosoma* and remains endemic in many tropical and subtropical regions, particularly in sub-Saharan Africa and parts of the Middle East [1,2]. Over 250 million people are infected with schistosomiasis worldwide [3], predominantly in impoverished tropical regions, and most cases occur in sub-Saharan Africa. Urogenital schistosomiasis caused by *S. haematobium* is endemic throughout Africa and parts of the Middle East [2], reflecting the distribution of *Bulinus* snail hosts. In Ethiopia alone, about 5.0 million people are infected and 37.5 million are at risk [3], underscoring the high regional burden.

The life cycle of *Schistosoma haematobium* involves freshwater snails (*Bulinus* spp.) as intermediate hosts. Miracidia hatch from eggs in human urine and infect snails, where they develop into cercariae. These cercariae are released into water and penetrate human skin during water contact [4]. Within the human host, the worms mature and settle in the venous plexuses of the bladder and ureters. Females lay eggs that become lodged in the bladder wall, provoking a granulomatous immune response that leads to inflammation, ulceration, fibrosis, and calcification of the urinary tract [2,5]. Consequently, patients often develop hematuria and other urinary tract pathologies. Chronic infection can cause obstructive uropathy, hydronephrosis, and increases the risk of squamous cell carcinoma of the bladder. In women, egg deposition in the genital tract causes female genital schistosomiasis (FGS), characterized by mucosal lesions, contact bleeding, vaginal discharge, and infertility [2,5–7].

Praziquantel remains the only effective anti-schistosome drug, typically administered in preventive chemotherapy programs targeting school-aged children and high-risk populations [8]. However, the drug does not prevent reinfection or affect immature worms, highlighting the need for integrated approaches, including health education, improved sanitation, and snail control [9].

The WHO’s 2021–2030 NTD roadmap explicitly aims to eliminate schistosomiasis as a public health problem by 2030. Meeting this goal requires highly sensitive diagnostics, especially as prevalence falls. The WHO target product profile for schistosomiasis diagnostics emphasizes the need to reliably detect low-prevalent (3–10%) infections [2]. The gold standard method, urine filtration microscopy, is simple but has low sensitivity in light or post-treatment infections [10]. Reagent-strip tests for hematuria provide a rapid and inexpensive screening option but cannot distinguish *S. haematobium* from other causes of urinary tract bleeding [11]. Molecular techniques such as polymerase chain reaction (PCR) and loop-mediated isothermal amplification (LAMP) offer high sensitivity, detecting as few as 1–5 eggs/10 ml urine, but require laboratory facilities and skilled personnel [12,13]. Antigen detection assays like the up-converting phosphor lateral-flow (UCP-LF) CAA test demonstrate excellent sensitivity (around 97%) in low-transmission settings but are not yet field-deployable in many endemic areas [14,15].

As prevalence and intensity decline, the sensitivity of conventional parasitological methods diminishes, leading to underestimation of infection rates [13]. Thus, there is a growing need for novel diagnostic approaches that combine high sensitivity and specificity with affordability and field adaptability.

Recent advances in artificial intelligence (AI) and deep learning have introduced new opportunities for diagnostic automation in parasitology. Convolutional neural networks (CNNs) can be trained to detect parasite eggs from digital microscopy images with high accuracy [16]. Smartphone-based platforms such as SchistoScope use AI to identify *S. haematobium* eggs directly from filtered urine images [17], while automated bench-top scanners (AiDx Assist) utilize deep learning models to quantify egg counts rapidly [18]. AI has also been applied in reading dipstick color changes for hematuria and proteinuria detection with over 97% accuracy [19].

Despite these advances, AI-based diagnostic studies for *S. haematobium* remain few and reported performance metrics vary widely. For instance, sensitivities range from 83% to 96% and specificities from 77% to 99%, depending on image acquisition, AI model, and reference standard used [16,20]. These inconsistencies require systematic evaluation to generate pooled estimates and guide implementation feasibility. Hence, the objective of this systematic review and meta-analysis was to systematically evaluate the diagnostic accuracy of artificial intelligence assisted tools for detecting *Schistosoma haematobium* infection.

## Methods

### Ethical statement

Not applicable.

### Search strategy and study selection

This systematic review and meta-analysis adhered to PRISMA guidelines [21], and was registered in PROSPERO (2025: CRD420251140737). We searched in PubMed/MEDLINE, HINARI, Epistemonikos, ScienceDirect, Google Scholar, and Google for studies published up to August 2025. Search terms included combinations of “Schistosoma haematobium” OR schistosomiasis) AND (“artificial intelligence” OR “machine learning” OR “deep learning” OR “AI”) AND (diagnosis OR “diagnostic accuracy” OR detect*. Gray literature and the reference lists of included studies were also reviewed to identify additional relevant articles. The search strategy was designed based on the PIRD (Population, AI-assisted diagnostic tools, Reference standard, Diagnosis of interest) framework. Literature searches were performed in all electronic databases on multiple dates between 5 and 31 August 2025.

Two reviewers (SD and TH) independently screened titles and abstracts to identify potentially eligible studies. The full text of selected articles was then assessed against the inclusion criteria. Any disagreements were resolved through discussion and consensus.

### Study selection

We included original studies evaluating an AI-assisted tool for detecting *S. haematobium* eggs in human urine samples, using microscopy or molecular diagnostics as the reference standard. Both cross-sectional and cohort study designs were eligible. Only peer-reviewed articles published in English were considered. We excluded review articles, editorials, letters, case reports, animal studies, and conference abstracts.

### Data extraction and quality assessment

From each included study, two reviewers independently extracted data using a standardized excel form. We collected information on first authors, year of publication, study setting and population, sample size, details of the AI-assisted diagnostic tools (device or algorithm), reference standard method, and diagnostic accuracy outcomes (numbers of true positives, false positives, true negatives, and false negatives). Discrepancies in data extraction were resolved by discussion. The methodological quality and risk of bias of each study were assessed using the QUADAS-2 (Quality Assessment of Diagnostic Accuracy Studies, version 2) tool, which evaluates bias in patient selection, AI-assisted diagnostic tools, reference standard, and flow and timing.

### Statistical analysis

Pooled accuracy of AI-assisted diagnostic tools for *Schistosoma haematobium* was analyzed against reference tests such as urine filtration microscopy or PCR using STATA version 14.1. The number of participants with true positive (TP), false positive (FP), false negative (FN), and true negative (TN) AI-assisted test results were used to calculate the sensitivity and specificity for each study as well as the overall summary estimates. Positive likelihood ratio (LR^+^), negative likelihood ratio (LR^−^), and diagnostic odds ratio (DOR) were also calculated. Subgroup analyses were performed based on reference standards, endemicity level, and AI algorithm type. To assess the ability of AI-assisted tools to discriminate participants with *S. haematobium* infection from non-infected individuals, a summary receiver operating characteristic (SROC) curve was generated, and the area under the curve (AUC) was interpreted as excellent (0.9-1.0), good (0.8-0.9), fair (0.7-0.8), poor (0.6-0.7), or failed (0.5- < 0.6). Between-study heterogeneity was evaluated using Cochrane’s Q test, and the I² statistic, with significant heterogeneity defined as I² > 50% and Q-test P < 0.10. Methodological quality of the included studies was assessed using the QUADAS-2 tool.

## Results

**Fig 1:**
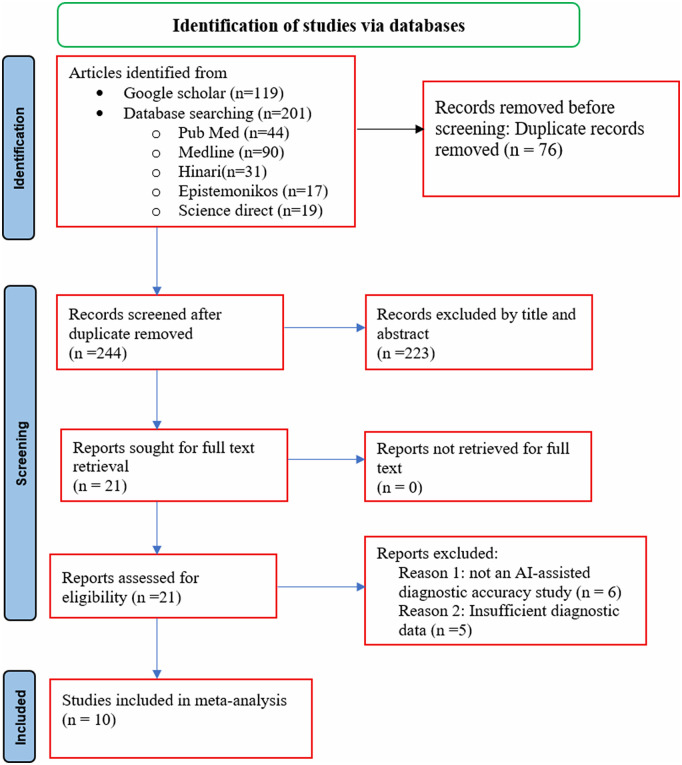
PRISMA flow diagram of the study selection process. The flowchart outlines the systematic identification, screening, and inclusion of studies investigating the accuracy of AI-assisted diagnostic tools for Schistosoma haematobium.

A total of 320 records were initially identified. After removing 76 duplicates, 244 titles and abstracts were screened, leading to the exclusion of 223 records. Twenty-one full-text articles were then assessed for eligibility, of which 11 were excluded (six were not AI-assisted diagnostic accuracy studies and five did not provide sufficient diagnostic data) (Fig 1). A total of 10 studies, comprising 15 datasets and involving participants, were included in this review (Table 1). Sample sizes ranged from 68 participants [20], to 869 in a large community-based study [22]. The studies were all conducted in sub-Saharan Africa, reflecting the endemic distribution of *Schistosoma haematobium*. Geographically, four studies were conducted in Nigeria [18,22–24], three in Côte d’Ivoire [17,20,25], and three in Gabon [18]. All included studies employed cross-sectional design. Four studies focused on school-aged children [17,18,25], while others were community-based studies that included participants aged five years and above [22,23] or mixed populations including preschool children, school-aged children, and adults [24]. Seven studies were conducted in moderate-to-high endemic settings such as Gabon and Nigeria [18,22,24], and three were conducted in lower prevalence communities, in Côte d’Ivoire [17,20]. This variation allows the applicability of AI-assisted diagnostics across different transmission intensities.

**Table 1:** Characteristics and diagnostic performance of included studies on AI-assisted tools for *Schistosoma haematobium* detection.

Author	Publication year	Country	Sample Size (n)	Study population	AI model (AI-assisted diagnostic tools)	Reference Standard	TP	FN	TN	FP	References
Armstrong *et al*	2022	Ghana &Côte d’Ivoire	205	School-aged children	SchistoScope (AI)	Urine filtration microscopy	45	4	142	14	[25]
Coulibaly *et al*	2022	Côte d’Ivoire	170	School-aged children	SchistoScope (AI)	Urine filtration microscopy	30	5	126	9	[17]
Makau-Barasa *et al*	2023	Nigeria	869	Community based and ≥5 years	AiDx Assist (automated)	Urine filtration microscopy	239	21	126	12	[22]
Meulah 4^a^ *et al*	2024	Gabon	339	School aged children, community	Schistoscope (AI)	Urine filtration microscopy	127	75	108	29	[18]
Meulah 4^b^ *et al*	2024	Gabon	339	School aged children, community	Schistoscope (AI)	Urine filtration microscopy	108	22	161	48	[18]
Meulah *et al*	2024	Gabon	798	School aged children, community	Schistoscope (AI)	Urine filtration microscopy	286	21	403	88	[18]
Meulah 7^a^ *et al*	2024	Gabon	349	School aged children, community	Schistoscope (AI)	Urine filtration microscopy	183	7	138	21	[18]
Meulah 7^b^ *et al*	2024	Gabon	349	School aged children, community	Schistoscope (AI)	Urine filtration microscopy	195	55	90	9	[18]
Meulah *et al*	2022	Nigeria	487	Preschool, School-aged children, adults	Schistoscope 5.0 (automated)	Urine filtration microscopy	145	21	157	164	[24]
Meulah *et al*	2025	Nigeria	398	Community based and ≥5 years	AiDx Assist (automated)	Urine filtration microscopy	239	21	126	12	[23]
Díaz de León Derby 11^a^ *et al*	2025	Côte d’Ivoire	68	Community based and ≥5 years	edge-tuned AI (BF)	Urine filtration microscopy	44	1	16	7	[20]
Díaz de León Derby 11^b^ *et al*	2025	Côte d’Ivoire	68	Community based and ≥5 years	edge-tuned AI (DF)	Urine filtration microscopy	38	7	20	3	[20]
Díaz de León Derby 13^a^ *et al*	2025	Côte d’Ivoire	375	School-aged children and adults	YOLOv8 (BF)	Urine filtration microscopy	38	12	313	12	[20]
Díaz de León Derby 13^b^ *et al*	2025	Côte d’Ivoire	375	School-aged children and adults	YOLOv8 (DF)	Urine filtration microscopy	41	9	313	12	[20]
Díaz de León Derby 13^c^ *et al*	2025	Côte d’Ivoire	375	School-aged children and adults	YOLOv8 (BF + DF)	Urine filtration microscopy	41	9	313	12	[20]

NB; ^a or b or c:^ indicates, in one study, two or three datasets are extracted.

Reference standards also varied. All studies (n = 10) used conventional urine filtration microscopy as the comparator [17,18,20,22–25]. Urine was consistently the diagnostic specimen across all studies.

Several AI-assisted platforms were assessed across the studies. These included the smartphone-based SchistoScope [17,18,25], the AiDx Assist, and fully automated diagnostic microscope [22,23], the Schistoscope 5.0, a field-deployable AI-enhanced microscope [24], and more advanced deep learning approaches such as YOLOv8 and edge-tuned AI [20].

### Data quality assessment

**Fig 2:**
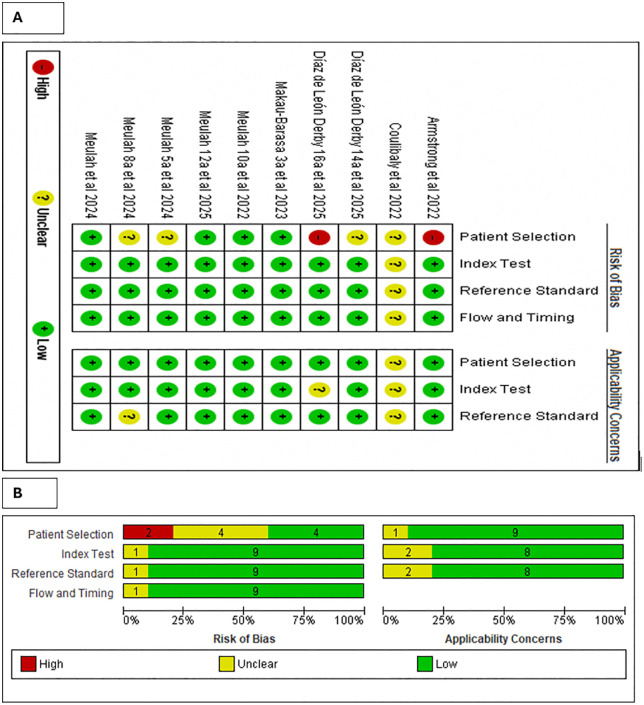
Summary of risk of bias and applicability concerns of included studies. Methodological quality was assessed using the Quality Assessment of Diagnostic Accuracy Studies (QUADAS-2) tool. **(A)** Risk of bias and applicability concerns individual studies across the four QUADAS-2 domains: patient selection, index test, reference standard, and flow and timing. **(B)** Summary plot showing the proportion of studies judged as low, high, or unclear risk of bias (left panel) and applicability concerns (right panel) for each domain.

The QUADAS-2 evaluation revealed that most studies demonstrated a low risk of bias across the domains of AI-assisted diagnostic tools, reference standard, and flow and timing. However, the patient selection domain showed some limitations, with two studies at high risk of bias and four rated as unclear, mainly due to non-random sampling or insufficient reporting of recruitment methods. Applicability concerns were low across all domains, although a small number of studies raised uncertainties in relation to the AI-assisted diagnostic tools (n = 2) and reference standard (n = 2). Overall, the evidence base is moderately robust, but pooled estimates should be interpreted with some caution due to potential bias arising from patient selection in a few studies (Fig 2).

### Diagnostic accuracy of AI-assisted diagnostic tools

**Fig 3:**
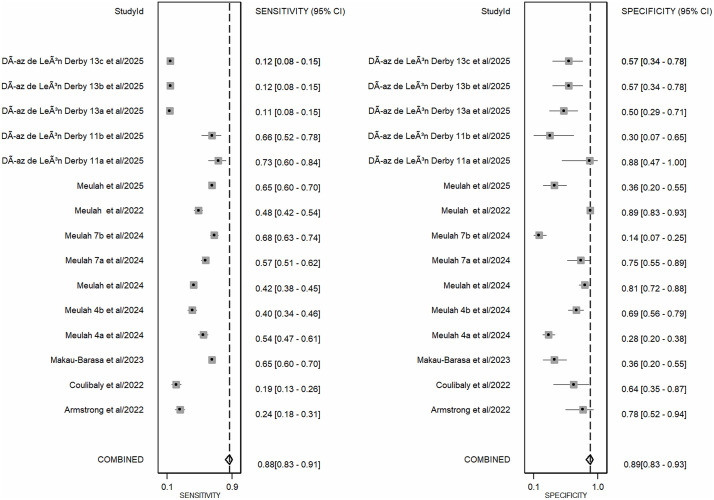
Forest plots of pooled sensitivity and specificity for AI-assisted diagnostic tools in detecting Schistosoma haematobium. The forest plots illustrate the diagnostic performance of AI-assisted tools across 15 included datasets. The left panel represents the Sensitivity 0.88 (95% CI: 0.83-0.91), and the right panel represents the Specificity 0.89 (95% CI: 0.83-0.93).

**Fig 4:**
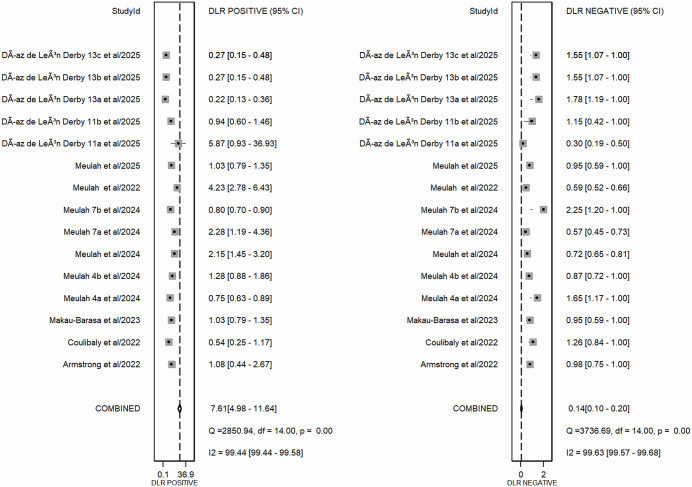
Forest plots for pooled positive and negative Diagnostic Likelihood Ratios (DLRs). The forest plots display the diagnostic performance of AI-assisted tools for Schistosoma haematobium across 15 datasets. The left panel shows the pooled Positive Diagnostic Likelihood Ratio (DLR^+^) of 7.61 (95% CI: 4.98-11.64), indicating a moderate-to-strong increase in the likelihood of infection given a positive AI result. The right panel shows the pooled Negative Diagnostic Likelihood Ratio (DLR^-)^ of 0.14 (95%CI: 0.10-0.20), suggesting that a negative AI result significantly reduces the probability of infection.

**Fig 5:**
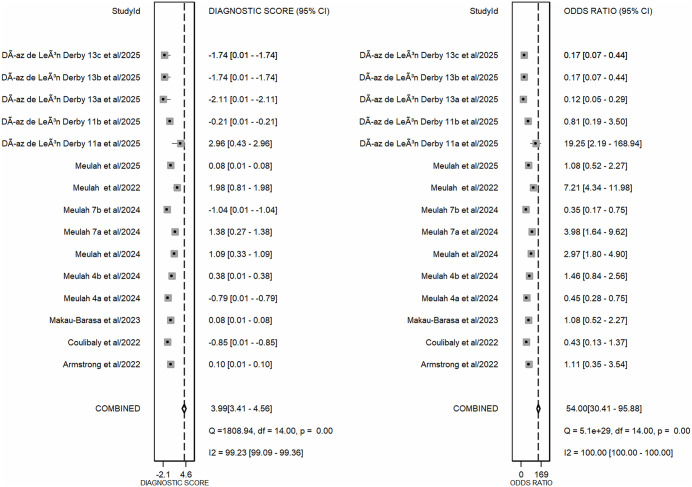
Forest plots for pooled diagnostic score and diagnostic odds ratio (DOR). The forest plots illustrate the overall diagnostic efficacy of AI-assisted tools for Schistosoma haematobium detection across 15 datasets. The left panel shows a pooled Diagnostic Score of 3.99 (95%CI: 3.41-4.56), indicating a high level of discrimination between infected and non-infected individuals. The right panel displays the pooled Diagnostic Odds Ratio (DOR) of 54.00 (95%CI: 30.41-95.88), which represents the ratio of the odds of a positive AI result in infected individuals compared to non-infected individuals.

**Fig 6:**
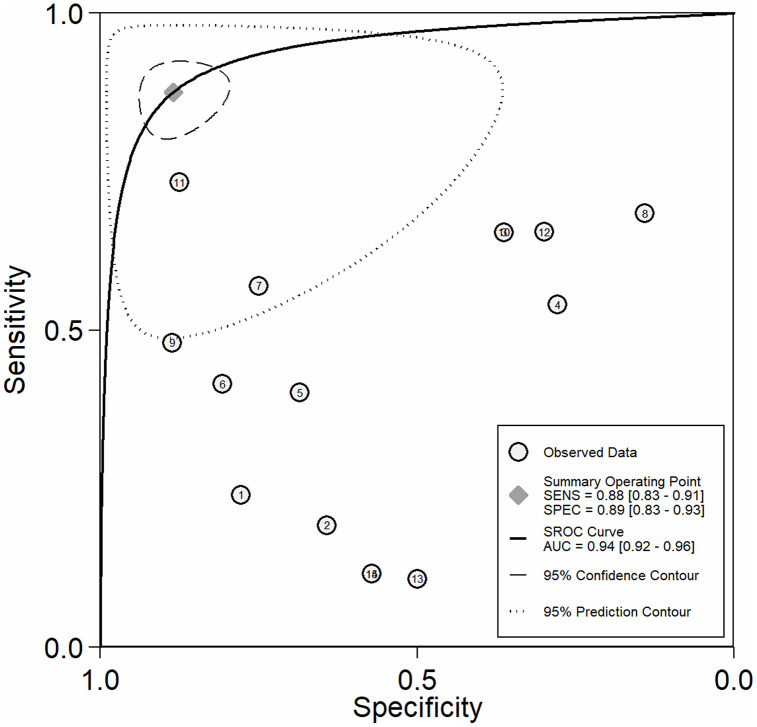
Summary receiver operating characteristic (SROC) curve for AI-assisted diagnostic tools. The SROC curve illustrates the overall diagnostic performance of AI-assisted platforms in detecting Schistosoma haematobium across 15 datasets. The Summary Operating Point (represented by the grey diamond) indicates a pooled sensitivity of 0.88 (95% CI: 0.83-0.91) and a pooled specificity of 0.89 (95% CI: 0.83-0.93). The Area Under the Curve (AUC) is 0.94 (95%CI: 0.92-0.96).

The meta-analysis, which combined all AI-assisted diagnostic tools (including SchistoScope, AiDx Assist, Schistoscope 5.0, and YOLO-based models), demonstrated that the AI-assisted diagnostic tools have high overall diagnostic accuracy. The pooled sensitivity across all included studies was 0.88 (95% CI: 0.83-0.91), while the pooled specificity was 0.89 (95% CI: 0.83-0.93) (Fig 3). The meta-analysis of diagnostic likelihood ratios yielded a pooled DLR^+^ of 7.61 (95% CI: 4.98-11.64) and a pooled DLR^-^ of 0.14 (95% CI: 0.10-0.20). These results suggest that AI-assisted diagnostic tools for *S. haematobium* are effective clinical filters (Fig 4). The meta-analysis revealed a high overall diagnostic efficacy for AI-assisted diagnostic tools. The pooled Diagnostic Odds Ratio (DOR) was 54.00 (95%CI: 30.41-95.88), suggesting that the odds of a positive test result are 54 times higher in individuals with *S. haematobium* than in those without. The pooled Diagnostic Score of 3.99 (95% CI: 3.41-4.56) also supports the high discriminatory power of these AI models. However, the wide confidence intervals and near-total heterogeneity (I^2^ = 100%) suggest that performance is highly dependent on the specific “Index Test” configuration (as seen in the QUADAS-2 assessment) (Fig 5). The overall performance of AI-assisted diagnostics was also evaluated using SROC analysis. The resulting Area Under the Curve (AUC) was 0.94 (95% CI: 0.92-0.96), which indicates that these tools have a high probability of correctly distinguishing between infected and non-infected individuals. The summary operating point reflects balanced performance, with both pooled sensitivity and specificity near 90% (Fig 6).

**Fig 7:**
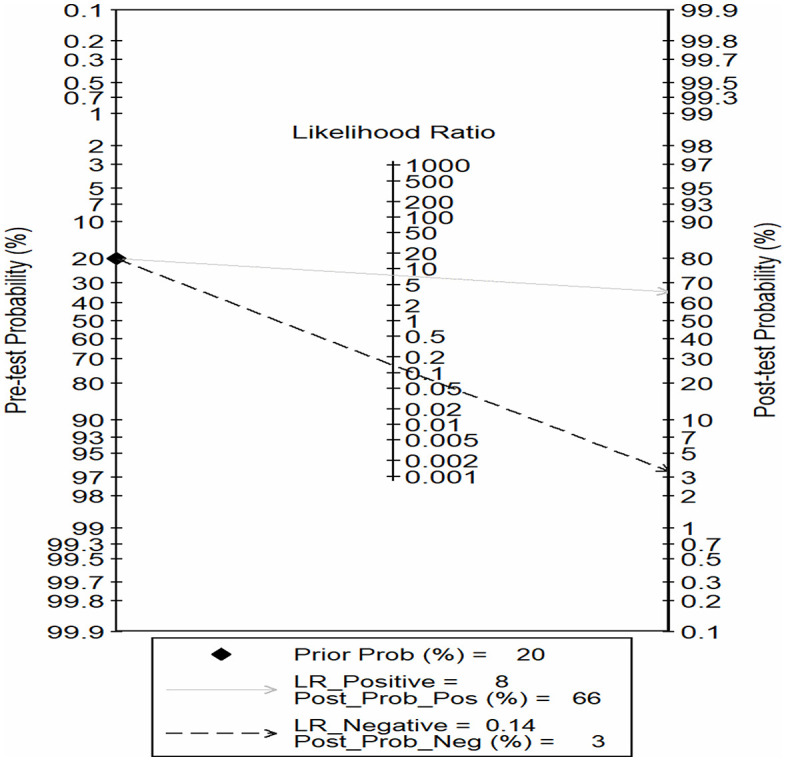
Fagan nomogram for the clinical utility of AI-assisted diagnostic tools. The Fagan nomogram displays the relationship between the pre-test probability, likelihood ratios, and post-test probability for Schistosoma haematobium detection. Assuming a baseline pre-test probability (prevalence) of 20%, a positive AI result (LR-Positive = 8) increases the post-test probability of infection to 66%. Conversely, a negative AI result (LR-Negative = 0.14) reduces the post-test probability of infection to 3%.

The clinical utility of AI-assisted diagnostic tools was evaluated using a Fagan nomogram. At an assumed regional prevalence (pre-test probability) of 20%, the application of the AI tool significantly shifted the probability of disease. A positive test result, with a pooled positive likelihood ratio (LR+) of 8, resulted in a post-test probability of 66%, indicating a substantial increase in the likelihood of *S. haematobium* infection. More notably, a negative test result, with a pooled negative likelihood ratio (LR-) of 0.14, reduced the post-test probability to 3% (Fig 7).

### Subgroup analysis and heterogeneity interpretation

#### By publication year.

The subgroup analysis done by publication year showed a statistically significant difference in diagnostic performance (p < 0.01), indicating that the year of study is a critical driver of the observed heterogeneity. For studies published in 2025 (n = 6), the pooled sensitivity was 88% (95% CI: 79%-93%) and the specificity was 93% (95%CI: 87%-92%), with a high level of statistical heterogeneity (I^2^ = 91%). Conversely, studies published prior to 2025 (n = 9) demonstrated an identical pooled sensitivity of 88% (95% CI: 81%-92%) but a lower specificity of 85% (95% CI: 76%-90%), accompanied by even greater heterogeneity (I^2^ = 98%) (Table 2).

**Table 2:** Subgroup analysis of pooled sensitivity and specificity of AI-assisted diagnostic tools for schistosomiasis, subgroup by publication year, endemicity, AI model type, and country setting.

Subgroup		No of datasets	Pooled sensitivity (95% CI)	Pooled specificity (95% CI)	Heterogeneity test (I^2^)	P-value
Publication year	2025	6	88% (79%-93%)	93% (87%-92%)	91%	<0.01*
	<2025	9	88% (81%-92%)	85% (76%-90%)	98%	<0.01*
Endemicity	Moderate to high endemic setting	8	88% (81%-93%)	83% (74%-89%)	97%	<0.01*
	Lower endemic setting	7	87% (79%-92%)	93% (88%-96%)	88%	<0.01*
AI-assisted diagnostic tools	SchistoScope (AI)	8	87% (79%-92%)	84% (74%-90%)	98%	<0.01*
	Another AI model	7	89% (82%-93%)	93% (88%-96%)	92%	<0.01*
Country setting	Gabon	5	86% (73%-94%)	83% (78%-87%)	95%	<0.01*
	Côte d’Ivoire	7	87% (79%-92%)	93% (88%-96%)	88%	<0.01*

Table 2 showed pooled estimates derived from random-effects meta-analysis. Sensitivity and specificity are presented with corresponding 95% confidence intervals (CI). Heterogeneity across studies within each subgroup was assessed using the I² statistic. A P-value <0.05 indicates statistically significant heterogeneity. P-values marked with an asterisk (<0.01*) indicate high heterogeneity.

Note: * statistically significant, NA-not applicable.

#### By endemicity level.

Across endemicity strata, AI-assisted diagnostic tools demonstrated similar sensitivity, with pooled estimates of 88% (81–93%) in moderate-to-high endemic settings and 87% (79–92%) in lower-endemic settings. However, specificity differed meaningfully between the two groups. Specificity was lower in moderate-to-high transmission settings (83% [74–89%]) compared with lower-endemic settings (93% [88–96%]). Heterogeneity remained high in both subgroups (I² = 88–97%, P < 0.01) (Table 2).

#### By AI-assisted diagnostic tools.

SchistoScope-based systems demonstrated a pooled sensitivity of 87% (79–92%) and specificity of 84% (74–90%). In comparison, other AI models showed slightly higher sensitivity (89% [82–93%]) and substantially higher specificity (93% [88–96%]). These findings suggest that while both categories of AI tools perform well in detecting true infections, non-SchistoScope AI systems may be more effective at minimizing false-positive outcomes. Nevertheless, heterogeneity was high across both categories (I² = 92–98%, P < 0.01), indicating wide variation in test performance across different study contexts and implementations (Table 2).

### Country setting

Performance also varied across country settings. Studies conducted in Gabon demonstrated a pooled sensitivity of 86% (73–94%) and specificity of 83% (78–87%), reflecting good diagnostic accuracy but with a moderate rate of false-positive classification. In contrast, studies from Côte d’Ivoire reported a similar sensitivity of 87% (79–92%) but a markedly higher specificity of 93% (88–96%). This suggests that AI-assisted diagnostic tools may achieve better specificity in some implementation environments than others. However, heterogeneity remained high in both countries (I² = 88–95%, P < 0.01), emphasizing persistent variability in AI performance across study designs and settings.

### Meta-regression analysis

The univariable meta-regression and subgroup analyses showed that differences in pooled sensitivity and specificity were partly explained by publication year, country setting, and AI model type. For sensitivity (left panel), the pooled estimates remained consistently high across subgroups, generally falling between approximately 0.85 and 0.95, indicating that AI-assisted tools reliably detect true schistosomiasis infections regardless of study characteristics. However, the AI model subgroup demonstrated a statistically significant effect on sensitivity, suggesting that some AI platforms perform better than others in identifying true-positive cases.

**Fig 8:**
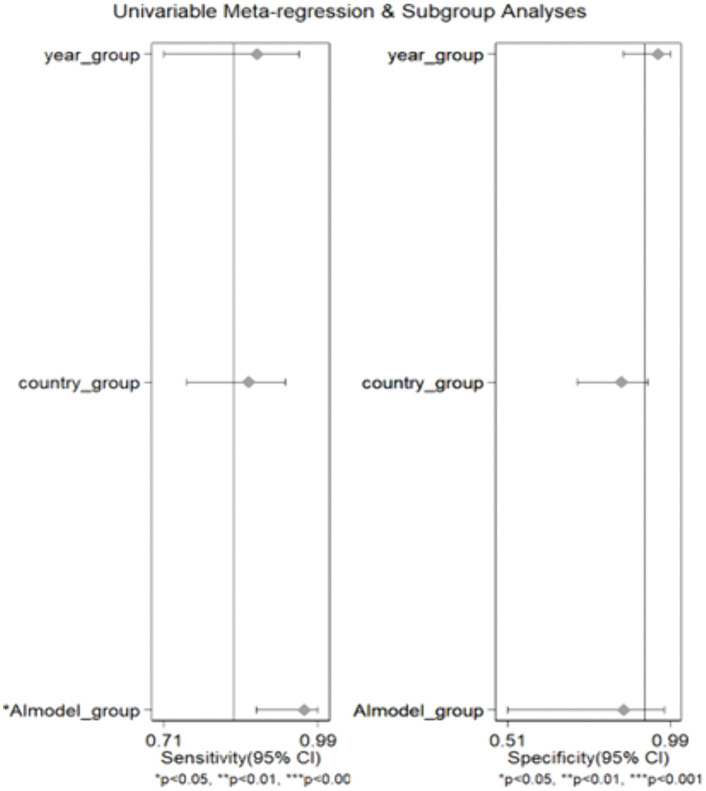
Univariable meta-regression and subgroup analyses evaluating the effect of study year, country setting, and AI model type on pooled sensitivity and specificity of AI-assisted diagnosis of schistosomiasis. Each point represents the pooled diagnostic performance estimate within the corresponding subgroup, with horizontal lines indicating the 95% confidence intervals (CI). The left panel presents subgroup effects on pooled sensitivity, while the right panel shows subgroup effects on pooled specificity. Subgroups include publication year category, country setting, and type of AI model.

For specificity (right panel), greater variability was observed across subgroups, with pooled values ranging from approximately 0.75 to >0.95. The AI model subgroup again showed a significant association with diagnostic performance, indicating that the choice of AI system influences the rate of false-positive results. Country setting also contributed to differences in specificity, with some geographic contexts showing higher accuracy for ruling out infection. Years of publication had less apparent influence on diagnostic accuracy, although slight improvements may be observed in more recent studies (Fig 8).

### Publication bias

**Fig 9:**
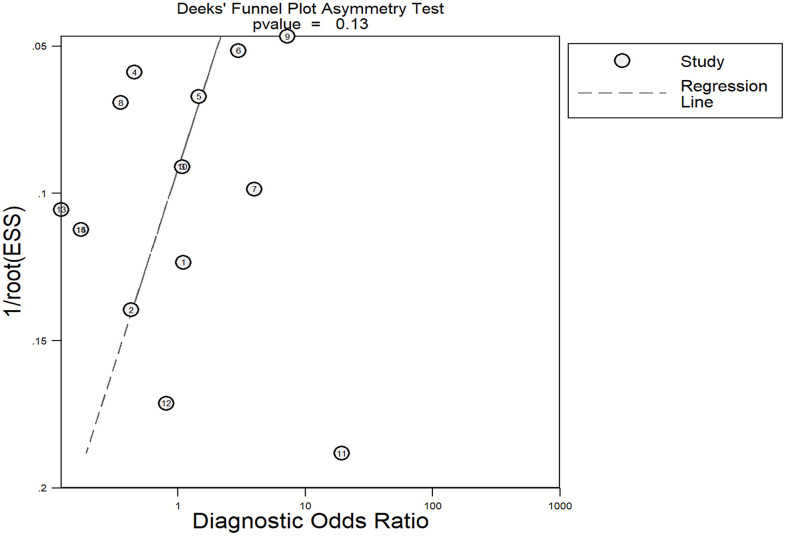
Deeks’ funnel plot asymmetrical test assessing small-study effects in the meta-analysis of AI-assisted schistosomiasis diagnostics. The figure presents Deeks’ funnel plot of diagnostic log odds ratios against the inverse root of the effective sample size. The regression line and its 95% confidence limits are shown. Asymmetry in the funnel plot indicates potential small-study effects or publication bias. The statistical significance of asymmetry was evaluated using Deeks’ regression test, with a P-value <0.10 indicating evidence of small-study effects.

Potential publication bias was assessed using Deeks’ funnel plot asymmetry test. Visual inspection of the funnel plot (Fig 9) showed a relatively symmetrical distribution of the studies on both sides of the regression line. Furthermore, the regression interception test yielded a p-value of 0.13, which is above the threshold of statistical significance (p > 0.05). Consequently, no significant evidence of publication bias was detected among the included studies, suggesting that the pooled estimates of diagnostic accuracy are unlikely to be skewed by the omission of small-scale or non-significant trials (Fig 9).

## Discussion

### Overall accuracy of AI-assisted diagnostics

Our meta-analysis indicates that AI-assisted image analysis tools have high diagnostic accuracy for *S. haematobium*. The pooled sensitivity was 0.88 (95% CI 0.83-0.91) and pooled specificity 0.89 (95% CI 0.83-0.93). These translate to a positive likelihood ratio (LR^+^) of 7.6 (95% CI 5.0-11.6) and a negative likelihood ratio (LR^–^) of 0.14 (95% CI 0.10-0.20). The diagnostic odds ratio (DOR) was 54 (95% CI 30–96), and the summary ROC area under the curve was 0.94. This means an AI-positive urine test in an area with moderate prevalence (pre-test probability 20%) would raise the post-test probability of infection to 66% (using an LR^+^=8), whereas an AI-negative result would drop it to 3% (LR^–^ = 0.14). This performance approaches or exceeds many target product profiles for schistosomiasis control. Díaz de León Derby *et al.* reported that a SchistoScope-based system achieved ≥81% sensitivity at 96.5% specificity meeting WHO “monitoring and evaluation” criteria for schistosomiasis diagnostics [20].

### Sources of heterogeneity across studies

Despite the above promising summary metrics, there was substantial heterogeneity (I² = 100%) in the pooled results, indicating variability among studies. Meta-regression showed that AI platform type, geographic setting, and transmission intensity explained much of this variation. In general, sensitivity remained uniformly high (0.85-0.95) across subgroups, but specificity varied widely (0.75-0.95). *SchistoScope* smartphone microscope had pooled sensitivity 87% but a lower specificity (84%), whereas non–SchistoScope platforms showed sensitivity 89% and higher specificity (93%). Similarly, country of evaluation affected specificity: studies in Côte d’Ivoire reported higher specificity (93%) than those in Gabon (83%). Endemicity also mattered: tests in moderate-to-high-transmission settings tended to have lower specificity (83%) than in low-endemic areas (93%). These differences likely reflect variations in sample egg burdens, examiner protocols, and device calibration. Notably, publication year had less impact; specificity was somewhat higher in very recent (2025) studies than earlier ones, showing improvements in algorithms or data quality over time.

### Features of AI diagnostic platforms

The AI tools themselves differ in design, which may contribute to heterogeneity. SchistoScope is a portable smartphone-based microscope that captures bright-field (BF) and dark-field (DF) images of filtered urine. It uses convolutional neural networks (YOLOv8) to detect eggs. For instance, Coulibaly *et al.* used the SchistoScope to screen in Côte d’Ivoire, reporting 85.7% sensitivity and 93.3% specificity relative to conventional microscopy [17]. Later work by Díaz de León Derby *et al.* trained separate YOLOv8 models on BF and DF images from the SchistoScope; combining contrasts improved detection, achieving 81% sensitivity at >96.5% specificity [26]. YOLOv8, a modern “You Only Look Once” object detector, provides high accuracy but requires more computational resources. By contrast, a leaner model like YOLOv5 may be preferable for deployment on low-power devices [26].

AiDx Assist is an AI-driven bench-top microscope designed for field use. Meulah *et al.* (2025) evaluated AiDx Assist in Nigeria and found *excellent* accuracy: semi-automated mode sensitivity 94.6% and specificity 90.6%, and fully automated mode 91.9% and 91.3%, respectively, for *S. haematobium* egg detection. This device uses integrated image capture and machine-vision algorithms to identify eggs on filters with minimal user input. Its high sensitivity meets WHO targets, but specificity <95% suggests some false positives when compared to light microscopy reference [23].

Schistoscope 5.0 is another automated digital microscope (developed by Oyibo *et al.*) with motorized scanning and autofocus. It produces whole-slide images and uses deep-learning segmentation (U-Net) to locate eggs. Oyibo *et al.* trained neural networks to segment *S. haematobium* eggs. Their results showed the Schistoscope’s imaging quality is comparable to a conventional microscope, and their AI algorithm could detect eggs in the presence of artifacts [27].

### Reference standard and gold-standard limitations

All included studies used urine filtration microscopy (examining eggs on filtered urine) as the reference standard. However, microscopy is acknowledged to be an *imperfect* gold standard. It is well established that urine filtration has limited sensitivity, especially for low-intensity infections [28,29]. Single-sample microscopy can miss light infections or miss eggs on days when ova output is low. Thus, assuming microscopy is 100% accurate is unrealistic. As noted by Shah *et al.* and others, “microscopic examination remains the gold standard with some limitations” [29]. Likewise, the WHO recommends multiple consecutive urine samples for more reliable diagnosis, but most studies use only one sample due to practicality [28].

Imperfect reference testing can bias accuracy estimates. If the reference misses true infections, some truly infected cases will be misclassified as “disease-negative” in the analysis. An AI tool that correctly identifies these cases would then be penalized as falsely positive, artificially lowering its measured specificity. Conversely, if AI misses an egg that microscopy found, this counts as a false negative, lowering sensitivity. In general, classical estimates of sensitivity/specificity assume a perfect gold standard; as a result, estimates can be distorted when this assumption fails. Indeed, our pooled metrics should be interpreted with caution: the true sensitivity of AI tools might be higher if compared to a flawless reference, and some observed false positives could reflect shortcomings of microscopy. Bayesian latent-class analyses (which explicitly account for imperfect tests) have shown that naively treating microscopy as perfect “can lead to biased accuracy estimates [28].

### Clinical utility: Likelihood ratios and Fagan’s Nomogram

We emphasize interpretation of likelihood ratios (LRs) to convey clinical utility. The pooled LR^+^ (7.6) indicates that a positive AI test makes infection 7–8 times more likely, while the pooled LR^–^ (0.14) indicates a decrease in probability if the test is negative. These LRs can be applied via Bayes’ theorem: pre-test probability (reflecting local prevalence or clinical suspicion) is converted to pre-test odds, multiplied by the LR, and then back transformed to a post-test probability [30]. A convenient visual aid is the Fagan nomogram, which graphically links pre-test probability, likelihood ratio, and post-test probability [30]. We used this approach to demonstrate that, even at low-to-moderate endemicity, the AI tests meaningfully change the diagnostic probability.

With a pre-test (prevalence) of 20%, our LR^+^ would yield a post-test probability of roughly 66% (significantly higher than 20%), whereas our LR^–^ would yield a post-test probability around 3%. This illustrates that AI-assisted microscopy can both *rule in* and *rule out S. haematobium* effectively. As Fagan’s framework, the impact of a diagnostic test depends crucially on the starting risk; in low-prevalence settings, even a high LR^+^ may not yield a definitive post-test (>95%), but it still significantly shifts probability. The graphical nomogram makes this transparent by showing how the line from pre-test (left axis) through the LR lands on post-test probability [30].

### Limitations

Although it is strengthened, this study is not beyond limitations. There are implementation challenges. Cost, durability, and training must be considered. Smartphone platforms (SchistoScope) require user training and stable lighting, while bench devices (AiDx, Schistoscope) may be more rugged but need periodic maintenance. Data connectivity for uploading images or updating AI models may also be limited. Importantly, any real-world program must establish quality control: e.g., periodically re-checking AI outputs against human readers. AI tools should ideally be integrated into target-product profiles with end-user feedback, as advocated in recent WHO surveys.

From a research perspective, these results show several priorities. First, prospective field trials are needed to confirm performance outside research settings, perhaps using composite reference standards to adjust for microscopy imperfections. Second, expanding AI algorithms to detect other NTD pathogens (e.g., *S. mansoni*, soil-transmitted helminths) could make them cost-effective multiplex tools. Third, refining “edge-tuning” approaches could standardize accuracy across diverse environments. Finally, further work should compare AI-assisted diagnosis with novel biomarkers to determine the best use-cases.

## Conclusion

The meta-analysis indicates that AI-assisted microscopy can achieve high accuracy in detecting S. haematobium. Pooled sensitivity was 0.88 (95%CI 0.83-0.91) and pooled specificity 0.89 (95%CI 0.83-0.93). The diagnostic odds ratio was 54 (95%CI 30–96). These values translate to clinically meaningful likelihood ratios: the pooled LR^+^ (7.6) and LR^–^ (0.14) imply that, at a 20% pre-test prevalence, a positive AI result raises post-test probability to 66%, whereas a negative result lowers it to 3%. In practical terms, AI screening would markedly increase the chance of correctly identifying infections and substantially reduce false negatives.

However, these findings must be interpreted considering key limitations. Between-study heterogeneity was extremely high (I² = 100%), reflecting variability in study populations, settings and AI implementations. All studies used conventional urine filtration microscopy as the reference standard, which is known to miss light or post-treatment infections. Because the “ground truth” may have been incomplete, we cannot conclude that AI tools truly overcome the gold-standard’s sensitivity gap. In other words, the reported accuracy may partly reflect concordance with an imperfect comparator, and further work (e.g., using composite or molecular reference tests) is needed to assess AI performance in low-intensity infections.

Despite these caveats, our results show robust performance of AI across diverse platforms. Both smartphone-based (SchistoScope) and automated bench-top systems (AiDx Assist, Schistoscope 5.0), as well as deep learning models like YOLOv8, achieved sensitivities and specificities near 90%. The summary ROC curve (AUC = 0.94) indicates that AI models consistently distinguish true infections from uninfected samples. This suggests that automated egg detection can be reliably deployed across endemic settings, reducing reliance on expert microscopists or laboratory infrastructure.

Finally, the public-health implications are promising. The WHO’s 2021-2030 NTD roadmap calls for eliminating schistosomiasis as a public-health problem by 2030, which requires highly sensitive, field-ready diagnostics in low-prevalence settings. AI-assisted tools, being portable and scalable, could help close this diagnostic gap. By improving case detection (and thus targeting treatment), these platforms may support elimination goals and reduce the burden of missed infections.

## Supporting information

S1 File Search strategy. Comprehensive electronic search strategies used across all databases, including search terms, Boolean operators, filters, and date limits applied to identify studies on AI-assisted diagnosis of *Schistosoma haematobium*.
(DOCX)

S1 Data Template data collection form. Standardized data extraction template used to collect study characteristics, diagnostic methods, reference standards, and accuracy outcomes from eligible studies.
(XLSX)

S2 Data Data extracted from included studies. Complete dataset extracted from all included studies, including study design, population characteristics, index tests, reference standards, and diagnostic accuracy measures.
(XLSX)

S1 Prospero Protocol PROSPERO protocol. Registered systematic review protocol detailing the study objectives, eligibility criteria, methodological approach, and planned analyses.
(PDF)

S1 PRISMA Checklist PRISMA-DTA checklist. Completed PRISMA-DTA checklist documenting adherence to reporting standards for diagnostic test accuracy systematic reviews and meta-analyses.
(DOCX)
