# Supplementary material for: Accuracy of AI-assisted diagnostic tools for Schistosoma haematobium: A systematic review and meta-analysis
Source: PLoS Negl Trop Dis. 2026 May 5;20(5):e0013703. doi: 10.1371/journal.pntd.0013703 (PMC13160431; doi:10.1371/journal.pntd.0013703)
Supplement: S1 File — Comprehensive electronic search strategies used across all databases, including search terms, Boolean operators, filters, and date limits applied to identify studies on AI-assisted diagnosis of Schistosoma haematobium. (DOCX) [file pntd.0013703.s001.docx]

**Diagnostic accuracy of AI- Assisted tools for Schistosoma haematobium: A systematic review and meta-analysis**

**1. Pub-Med N=44**

**08/28/2025**

(("Schistosoma haematobium"[MeSH] OR "Schistosoma haematobium" OR "urinary schistosomiasis" OR "blood fluke" OR "schistosome infection") AND ("Artificial Intelligence"[MeSH] OR "AI" OR "machine learning" OR "deep learning" OR "neural network*" OR "computer-assisted" OR "computer aided" OR "automated diagnosis" OR "algorithm*") AND ("diagnosis"[MeSH] OR "diagnostic accuracy" OR "sensitivity" OR "specificity" OR "predictive value*" OR "ROC curve*" OR "performance" OR "detection")) AND ("humans"[MeSH] OR human))

Filter: 10 years later

**2. Hinari N=31**

**08/28/2025**

("Schistosoma haematobium" OR "urinary schistosomiasis" OR "blood fluke" OR "schistosome infection") AND ("artificial intelligence" OR AI OR "machine learning" OR "deep learning" OR "neural network*" OR "computer-assisted" OR "computer aided" OR "automated diagnosis" OR algorithm*) AND ("diagnosis" OR "diagnostic accuracy" OR sensitivity OR specificity OR "predictive value*" OR "ROC curve *" OR performance OR detection)

Filter: 10 years later, Journal article

**3. Epistemonikos N=17**

**08/28/2025**

(title:("Schistosoma haematobium" OR "urinary schistosomiasis" OR "blood fluke" AND "artificial intelligence" OR AI OR "machine learning" OR "deep learning" OR "neural network" OR "computer-assisted" OR "automated diagnosis" OR algorithm AND diagnosis OR "diagnostic accuracy" OR sensitivity OR specificity OR "predictive value" OR "ROC curve" OR performance) OR abstract:("Schistosoma haematobium" OR "urinary schistosomiasis" OR "blood fluke" AND "artificial intelligence" OR AI OR "machine learning" OR "deep learning" OR "neural network" OR "computer-assisted" OR "automated diagnosis" OR algorithm AND diagnosis OR "diagnostic accuracy" OR sensitivity OR specificity OR "predictive value" OR "ROC curve" OR performance))

Filter: Publication year=Last 10 years, Publication type=primary research

**4. Medline N=90**

**08/28/2025**

("Schistosoma haematobium" OR "urinary schistosomiasis" OR "blood fluke") AND ("artificial intelligence" OR AI OR "machine learning" OR "deep learning" OR "neural network *" OR "computer-assisted" OR "computer aided" OR "automated diagnosis" OR algorithm*) AND (diagnosis OR "diagnostic accuracy" OR sensitivity OR specificity OR "predictive value *" OR "ROC curve*" OR performance OR detection)

**5. Google scholar N=119**

**08/28/2025**

"Schistosoma haematobium" OR "urinary schistosomiasis" OR "blood fluke" AND "artificial intelligence" OR AI OR "machine learning" OR "deep learning" OR "neural network" OR "computer-assisted" OR "automated diagnosis" OR algorithm AND diagnosis OR "diagnostic accuracy" OR sensitivity OR specificity OR "predictive value" OR "ROC curve" OR performance

**6. Science Direct N=19**

**08/28/2025**

("Schistosoma haematobium" OR "urinary schistosomiasis") AND ("artificial intelligence" OR AI OR "machine learning" OR "deep learning") AND (diagnosis OR "diagnostic accuracy")

Filter: Last 10 years, Article type= Research articl
