# Supplementary material for: Accuracy of AI-assisted diagnostic tools for Schistosoma haematobium: A systematic review and meta-analysis
Source: PLoS Negl Trop Dis. 2026 May 5;20(5):e0013703. doi: 10.1371/journal.pntd.0013703 (PMC13160431; doi:10.1371/journal.pntd.0013703)
Supplement: S1 Prospero Protocol — Registered systematic review protocol detailing the study objectives, eligibility criteria, methodological approach, and planned analyses. (PDF) [file pntd.0013703.s004.pdf]

PROSPERO protocol: Diagnostic accuracy of AI-assisted tools for *Schistosoma haematobium*: A systematic review and meta-analysis.

1. Review title

Diagnostic accuracy of AI-Assisted tools for *Schistosoma haematobium*: A systematic review and meta-analysis

2. Objective

To systematically review and meta-analyze the diagnostic accuracy (sensitivity and specificity) of AI-assisted tools, including smartphone-based microscopes and automated optical devices, for detecting *S. haematobium* infection in endemic settings.

3. Review Context / Background

Urogenital schistosomiasis, caused by *Schistosoma haematobium*, remains a significant public health problem in sub-Saharan Africa. Conventional diagnostic methods, such as urine filtration and microscopy, often miss light infections and require trained personnel and laboratory infrastructure. Recent AI-assisted tools, including smartphone-based microscopes and automated optical devices, promise faster, more accessible, and potentially more accurate detection at the point of care. However, evidence on their diagnostic performance is scattered and variable. This review will synthesize available studies to evaluate the sensitivity and specificity of AI-assisted tools for *S. haematobium*, providing guidance for their use in surveillance and control programs.

4. Participants / Population

- ✓ Individuals of all ages living in *S. haematobium*-endemic regions.
- ✓ No restriction on sex, ethnicity, or health status.

5. Interventions (AI MODEL)

- ✓ AI-assisted diagnostic tools for detecting *S. haematobium*, including but not limited to:
    - ✓ Smartphone-based microscopes
    - ✓ Automated optical devices with AI-based egg detection

- ✓ Digital imaging systems with machine learning algorithms

6. Comparators (reference standards)

- ✓ Conventional diagnostic methods:
  - ✓ Urine filtration and microscopy
  - ✓ Reagent strips for macrohematuria
  - ✓ Molecular or antigen detection (where available)

7. Outcome measures

Primary outcome:

- ✓ Diagnostic accuracy measures: sensitivity, specificity of AI-assisted tools compared to reference standards.

8. Study design

- ✓ Diagnostic accuracy studies (cross-sectional, cohort, or case-control)
- ✓ Field evaluations of AI-assisted tools for *S. haematobium*
- ✓ Both published and unpublished studies, including conference abstracts and reports

9. Search strategy

- ✓ Databases: PubMed, MEDLINE, Epistemonikos, Hinari, Science Direct,
- ✓ Other source: Google Scholar
- ✓ Keywords and MeSH terms:

  (("Schistosoma haematobium"[MeSH] OR "Schistosoma haematobium" OR "urinary schistosomiasis" OR "blood fluke" OR "schistosome infection") AND ("Artificial Intelligence"[MeSH] OR "AI" OR "machine learning" OR "deep learning" OR "neural network*" OR "computer-assisted" OR "computer aided" OR "automated diagnosis" OR "algorithm*") AND ("diagnosis"[MeSH] OR "diagnostic accuracy" OR "sensitivity" OR

"specificity" OR "predictive value*" OR "ROC curve*" OR "performance" OR "detection")) AND ("humans"[MeSH] OR human))

- ✓ No language restrictions; studies from inception to present

10. Data extraction

- ✓ Two reviewers will independently screen titles, abstracts, and full texts.
- ✓ Extracted data:
  - ✓ Study characteristics: author, year, country, setting
  - ✓ Participant demographics: age, sex, infection prevalence/intensity
  - ✓ Index test details: device type, AI algorithm, sample preparation
  - ✓ Reference standard
  - ✓ 2by 2 data: true positives (TP), false positives (FP), false negatives (FN), true negatives (TN)

11. Risk of bias (Quality assessment)

- ✓ QUADAS-2 tool for diagnostic accuracy studies
- ✓ Domains assessed: patient selection, index test, reference standard, flow, and timing

12. Data synthesis

- ✓ Quantitative meta-analysis using bivariate random-effects models for pooled sensitivity and specificity
- ✓ Summary receiver operating characteristic (SROC) curves
- ✓ Subgroup analyses:
  - ✓ Device type
  - ✓ Field vs. laboratory settings
- ✓ Heterogeneity assessed using I^2 statistic and visual inspection of forest plots

13. Analysis of subgroups (sensitivity analysis)

- ✓ Sensitivity analysis excluding studies with high risk of bias
- ✓ Subgroup analysis by age group, geographic region, and AI algorithm type

14. Dissemination plan

- ✓ Findings will be published in a peer-reviewed journal and presented at relevant conferences.
- ✓ Results will inform policymakers, program implementers, and researchers on the utility of AI-assisted diagnostic tools in schistosomiasis control programs.

15. Funding (Conflicts of interest)

- ✓ Funding: none declared
- ✓ Conflicts of interest: none declared

16. PROSPERO registration details

- ✓ Anticipated start date: 09/04/2025
- ✓ Anticipated completion date: 10/10/2025
